# Triazole-Modified Nucleic Acids for the Application in Bioorganic and Medicinal Chemistry

**DOI:** 10.3390/biomedicines9060628

**Published:** 2021-05-31

**Authors:** Dagmara Baraniak, Jerzy Boryski

**Affiliations:** Institute of Bioorganic Chemistry, Polish Academy of Sciences, Noskowskiego 12/14, 61-704 Poznan, Poland; jboryski@ibch.poznan.pl

**Keywords:** click chemistry, 1,2,3-triazoles, backbone modifications, triazole-linkage, clickable nucleosides and nucleotides, triazole-modified oligonucleotides, (TL)DNA, (TL)RNA, (TL)LNA, (TL)BNA, (TL)PNA, (TL)quadruplexes, non-coding RNA, synthetic genes

## Abstract

This review covers studies which exploit triazole-modified nucleic acids in the range of chemistry and biology to medicine. The 1,2,3-triazole unit, which is obtained via click chemistry approach, shows valuable and unique properties. For example, it does not occur in nature, constitutes an additional pharmacophore with attractive properties being resistant to hydrolysis and other reactions at physiological pH, exhibits biological activity (i.e., antibacterial, antitumor, and antiviral), and can be considered as a rigid mimetic of amide linkage. Herein, it is presented a whole area of useful artificial compounds, from the clickable monomers and dimers to modified oligonucleotides, in the field of nucleic acids sciences. Such modifications of internucleotide linkages are designed to increase the hybridization binding affinity toward native DNA or RNA, to enhance resistance to nucleases, and to improve ability to penetrate cell membranes. The insertion of an artificial backbone is used for understanding effects of chemically modified oligonucleotides, and their potential usefulness in therapeutic applications. We describe the state-of-the-art knowledge on their implications for synthetic genes and other large modified DNA and RNA constructs including non-coding RNAs.

## 1. Introduction

### 1.1. A Brief Introduction to DNA and RNA World

The natural nucleic acids, DNA and RNA, are exquisitely suited to store and propagate genetic information [1,2]. Nucleic acids are unique among biopolymers, because of their high-water solubility together with denaturing and refolding abilities [3]. DNA is an enormously intricate yet simple molecule. Its beauty is hidden in a stable double-stranded helix, that is guarded in the cell nucleus for storing genetic material and because of its self-replication abilities. In turn, remarkable RNA most often is a single-stranded molecule, but it could adopt different structures and functions in the cell. It can mediate the heritable storage, recognize specific targets (aptamers), and even catalyze reactions [4]. RNA molecules are generally less stable and are quickly degraded by intracellular enzymes, hence the great need for chemical modifications. Thus, understanding and cognition of their capabilities such as direct protein synthesis and modulation of gene expression allow us to understand how life works [5]. Likewise, it is worth to notice that in all DNA and RNA technologies the Watson–Crick base pairs are responsible for the information content of all living systems [6,7,8,9]. Nowadays, RNA has become a focus of attention because it turned out that various RNAs are valuable tools in molecular biology and medicine. These molecules, including all classes of ncRNAs, are used in the design and development of drugs for fighting cancers, viruses, and other diseases at the nucleic acids level.

### 1.2. Non-Coding RNA

**Figure 1:**
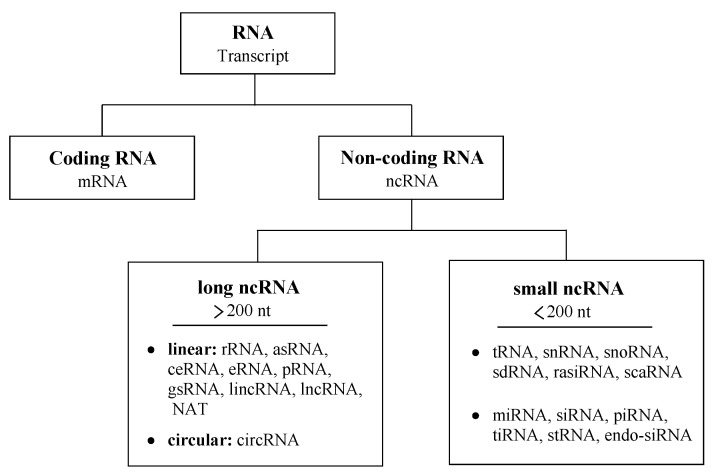
Diagram presenting the RNA world.

In the world of science, the key role for regulating life processes is ascribed to proteins. Since the genetic information about them is encoded in 2′-deoxyribonucleic acid (DNA), it found itself on the podium of fame. An unappreciated ribonucleic acid (RNA) stood in its shade—a silent hero with many faces. From the central dogma of molecular biology, it can be seen that the flow of genetic information, which takes place from DNA through RNA to protein, involves a molecule of RNA, and exactly the messenger RNA (mRNA). It follows that the mRNA molecule is coding RNA in transcription, because it rewrites information from DNA about proteins. A further stage is yet involved: ribosomal RNA (rRNA) and transfer RNA (tRNA). The first one acts as a scaffold, and the second one is a courier in the delivery of amino acids that are linked into long chains to form a protein. So, in translation, these two RNAs belong to the group of ncRNAs as they do not encode any protein sequence [10,11]. We know that the proteins-coding genes constitute only 2–3% of the entire human genome and the remaining 97–98% (wrongly called “junk” DNA) [12], they are sequences responsible for the formation of a numerous and diverse group of ncRNA molecules (Figure 1) [13,14].

More than 40% of the human genome is transcribed as ncRNA, what causes that chemical diversity in RNAs is huge. The above-mentioned rRNA and tRNA are molecules that occur in all cells, because they control the basic processes, therefore minor disturbances in the function can cause serious diseases. The fact is that the RNA world is rich—it can be more independent, which is evidenced in the discovery of ribozymes, and that has become a milestone in molecular biology [15]. It has been proved that RNA molecules are capable of carrying out the reaction—editing mRNA, modifying immature tRNA, or participating in the synthesis of ribosomes. But they can also act as diagnostic tools, *e.g.*, small nuclear RNA (snRNA) or small nucleolar RNA (snoRNA) [16,17]. In turn, regulatory microRNAs (miRNAs) control gene expression, and are also involved in brain development and nerve cell specialization [18]. Disorders of these molecules lead to the development of tumors, including brain gliomas [19,20], gastric cancer [21], or breast cancer [22]. Therefore, research is ongoing to develop anti-cancer strategies based on miRNAs [23], *e.g.*, preparation of synthetic oligonucleotides complementary to these miRNA molecules, of which overexpression causes brain gliomas [24,25]. Summarizing and “encoding a non-coding” RNAs, they are thought to contribute in structural functions—(a) they build a framework for proteins with which they occur in a complex, e.g., rRNA; (b) form antisense threads (“guides”)—complementary to target RNAs, form Watson–Crick pairs with them, *e.g.*, miRNA, siRNA, piRNA; (c) catalytic (“ribozymes”)—they catalyze biochemical reactions, *e.g.*, hammerhead, RNaseP; (d) regulatory functions—regulate gene expression, *e.g.*, riboswitches; (e) control epigenetic phenomena [26,27]. In the treatment of human diseases, miRNA are regulators of endogenous gene expression, and thus biomarkers in cancer [28]. In turn, siRNAs are directed against oncogenes, but also in the treatment of viral diseases (HIV, HCV, influenza virus). We see that the huge functional diversity of these molecules allows us to better understand gene expression, create new therapeutic strategies and better develop diagnostic methods for many diseases [29,30].

### 1.3. Click Chemistry

**Figure 2:**
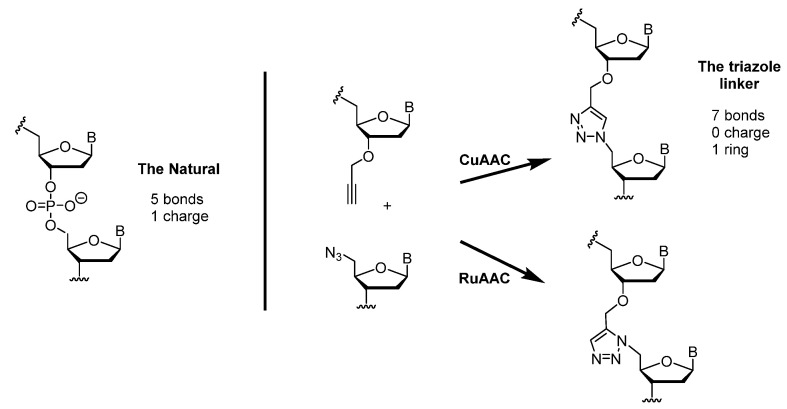
The comparison of the natural internucleotide linkage with a 1,4- or 1,5-disubstituted 1,2,3-triazole linker.

The concept of organic chemistry, introduced by Sharpless and Meldal, named *click chemistry*, lies in using simple and efficient reactions designed to bind various compounds in an easy way [31,32]. Simple reaction conditions and readily available reagents lead to the formation of stable products, which very often exhibit some unique biological properties. Among the click reactions, the most important role is played by the catalyzed 1,3-dipolar Huisgen cycloaddition involving organic azides and terminal alkynes, and leading to products of 1,2,3-triazole structures (Figure 2). Click chemistry, especially the Huisgen reaction catalyzed by copper(I) or ruthenium(II) ions (in short: CuAAC or RuAAC, respectively), arouses great interest among scientists due to its simplicity and convenient conditions [33,34,35]. In recent years, hundreds of scientific works describing applications of click chemistry have been published, including extensive research papers. For us, the works in the field of biological chemistry are of particular importance, pertaining to synthesis of small molecules—clickable nucleosides and nucleotides [36,37,38,39,40], dimers derivatives—canonical [41,42] and non-canonical [43] linked dinucleosides, and modified oligodeoxynucleotides [44,45,46]. Among the discussed topics, other important applications can be listed, such as: receiving fluorescent markers [47,48,49,50,51,52,53,54], ligand design [55], crosslinking duplexes [56,57,58], labeling [59,60,61,62,63], conjugates [64,65,66], surface modification [67], artificial molecular machines [68], nanomaterials, and nanostructures [69,70,71].

Exploiting the CuAAC and RuAAC tools also opens access to complex architecture in biomedicinal science, for example: drug discovery [72,73,74,75], triazole-linked analogues of oligonucleotides [76], and therapeutic nucleic acids (“clicking” genes) [77,78,79] with different arrangement of the triazole linker [80], and enables gene editing by CRISP-Cas9 method [81,82,83,84].

An additional impact on the science is described in works related to aptamers and chimeric biomolecules [85,86,87,88,89,90], peptides and proteins [91,92,93,94,95,96,97,98,99,100], antibody–oligonucleotide conjugates [101,102,103,104,105,106], vaccines [107], or building blocks of viruses [108]. There are many reviews discussing modified nucleic acids as therapeutic agents [109,110,111]. This presents how great is the need for oligonucleotides with improved properties including enhanced recognition and binding to RNA, duplex DNA, and proteins. This results in the development of new potent antisense and antigen agents [112,113,114]; however, only extremely accurate chemistry and biochemistry can make genes and even genomes. Every scientist knows how it can be frustrating because of the cost of synthetic genes. Additionally, the standard chemical approach to synthesize oligonucleotides, namely the phosphoramidite chemistry cycle, is limited to around 100-base building blocks; beyond that length they are very difficult to make [115]. Therefore, people all over the world are trying to find a way to make them more affordable and on a larger scale. Herein, the development of various chemical methods can come to aid the nucleic acid synthesis. One of them is so-called click chemistry, that assembles modified nucleoside and nucleotide building blocks using entirely chemical methods [116]. Here, we explore recent discoveries in the synthesis and functional research of triazole-modified oligonucleotides with a view towards therapeutic applications. Using click reaction that creates a triazole, we receive a stable linker replacing the internucleotide bond [117,118,119]. With the need for better gene synthesis, all innovative ideas attract a great deal of attention [120,121,122,123].

## 2. Triazole-Modified Oligonucleotides

Chemical modifications of oligonucleotides (ONs) came to prominence in the early 1990s with the advent of the antisense approach to control gene expression [124,125]. Antisense strategy as a part of controlling gene expression is a fast-growing research area. Naturally occurring oligonucleotides are unsuitable for that purpose because of the low stability towards nucleolytic degradation and poor cell penetration due to their polyanionic structure. These problems could be overcome by the chemical modification of oligonucleotides. ONs can be used in antisense or triple helix therapy to inhibit RNA translation or DNA transcription. One of the most radical modifications brought to the familiar backbone is the complete substitution of the phosphodiester bridge, to achieve stronger affinity for the nucleic acid target, and/or enhanced resistance to nucleases, or improve membrane permeability and cellular uptake. Here, the catalyzed Huisgen reaction appears promising as a way to generate non-natural oligonucleotides, that cannot be biologically degraded but can be used as biological tools binding to native DNA/RNA. Thus, oligonucleotide analogues with triazole internucleotide linkages continue to attract significant attention on medical applications for gene silencing such as antisense, antigen [126,127], and siRNA [128,129,130,131,132] interference.

### 2.1. (TL)DNA Skeleton

**Figure 3:**
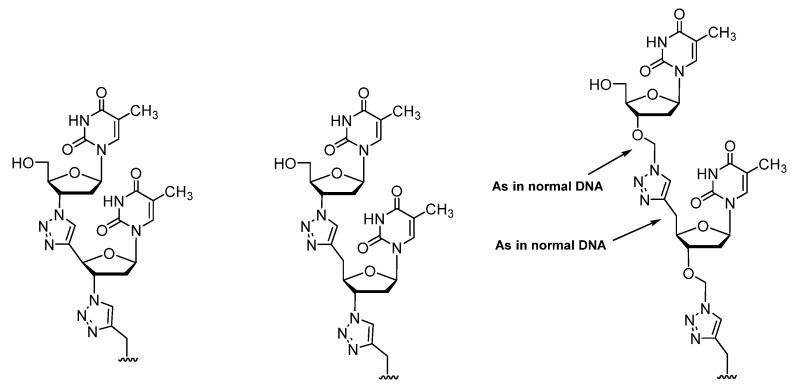
Triazole DNA template.

DNA is the molecule of life, because the information about the structure and functioning of the organism is encoded in it. Therefore, its structure and possibilities of “improvement” have always intrigued scientists all over the world (Figure 3). DNA usually forms a duplex, in other words double-stranded structure. Some elements of nucleic acids can take unusual structures, *e.g.*, in the case of an excess of guanine base. Then, a four-strand nucleic acid structure called G-quadruplex is formed, characterized by high plasticity compared to the standard double-stranded form, which translates into their important biological functions.

Studies of the properties of modified oligonucleotides led to the development of unnatural oligomers with huge therapeutic potential, especially for the treatment of diseases characterized by the expression of unwanted genes. The most promising of such analogues are those exhibiting high binding affinity toward native DNA/RNA, since they may prove to be efficient antisense or siRNA agents [133]. That is why a comprehensive analysis of the relation between the length of triazole internucleotide linkage and duplex stability is essential [34,76,134,135]. Therefore, it is logical that researchers turned their attention toward the possible benefits of innovative and new synthetic approaches, such as the CuAAC methods [33].

**Figure 4:**
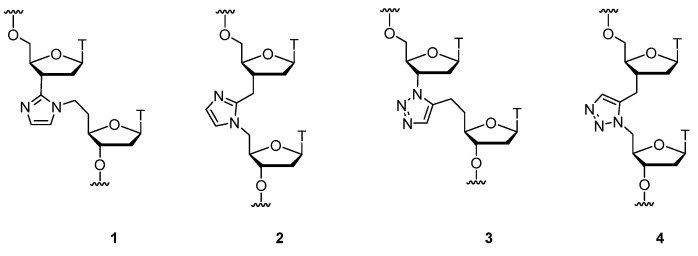
Thymidine dinucleoside analogues with imidazole and triazole modified backbones.

The first works which describe the replacement of the phosphodiester linkage in oligonucleotides by heterocycles have been reported by von Matt *et al.* [136,137]. This research presents synthesis of thymidine dinucleoside analogues with imidazole and triazole modified backbones before the era of click chemistry (Figure 4). Authors used a thermal cycloaddition to generate T-T dimers wherein triazole units were inserted (analogues of types **3** and **4**). The two communications described the detailed chemical synthesis of these compounds with an overview of the introduction into oligodeoxyribonucleotides strand in a DNA/RNA heteroduplex and evaluation of the influence of polarity and basicity of the modified backbone structure on RNA binding affinity, as well as thermodynamic properties. Only dimer **3** could oligomerize, while all attempts to incorporate the dimer **4** were completely unsuccessful. The backbone replacements evaluated lead to a destabilization of the duplexes formed by all modified sequences with their RNA complements. Only dimers of type **1** and **3** showed no significant improvement of duplex stabilization. In all instances, the incorporation of heterocyclic backbone modifications leads to reduction of the thermal stability of duplexes.

**Figure 5:**
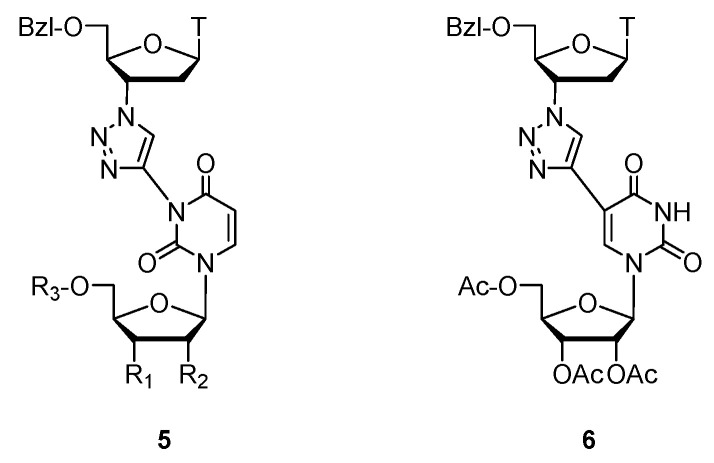
*N*-3 or *C*-5 branched nucleoside dimers containing a 1,2,3-triazolyl linkage.

Another type of coupling between two pyrimidine analogues has been accomplished by Lazrek *et al.* (Figure 5) [138]. The novel branched nucleoside dimers containing a 1,2,3-triazolyl linkage have been synthesized using 1,3-dipolar cycloaddition of *N*-3 or *C*-5 acetylene nucleosides with 3′-azido-3′-deoxythymidine (AZT). The coupling reactions under reflux always provide a mixture of the two possible 4- and 5-substituted isomers. As the authors suggest, such modified nucleosides may be of interest as potential candidates for nucleoside-based therapeutics.

**Scheme 1:**
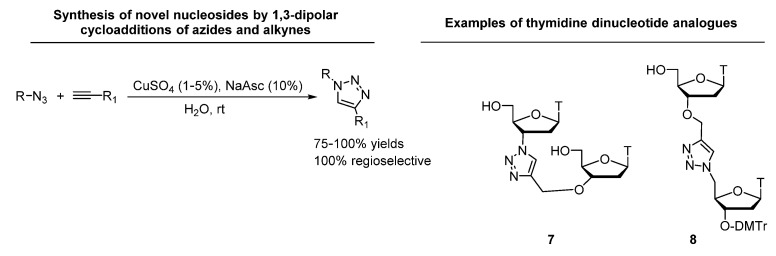
One of the first examples of AZT dimer analogues obtained by CuAAC.

Because of the important role of AZT in chemotherapy scientists are constantly looking for its valuable analogues. Also in this case, an inestimable support is given by click chemistry [33,55,79]. Using this approach, the Torrence group has reported the first synthesis of nucleoside 1,2,3-triazole derivatives using CuAAC [139] (Scheme 1). For this reaction, Sharpless’s original conditions using NaAsc to reduce Cu(II) to Cu(I) in water are employed. Dinucleosides **7** and **8**, in which two thymidines are linked by triazole, have been synthesized by reacting AZT or 5′-azidothymidine with 3′-*O*-propargylthymidine in excellent yields. Generally, 1% CuSO_4_ catalyst is enough to carry out this reaction. The reaction proceeds to completion at ambient temperature within 12 h. All novel thymidine analogues with a broad scope of modifications have been examined for their antiviral activity, but they have been found to be inactive against 13 viruses.

**Scheme 2:**
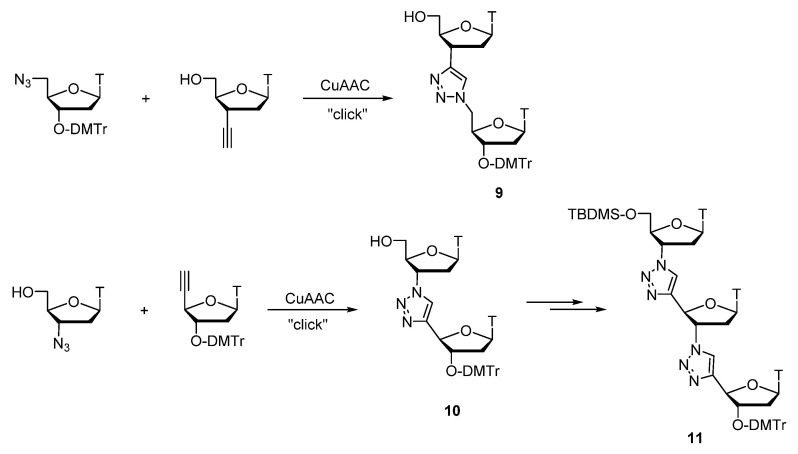
Synthesis of non-natural dinucleotides and trinucleotide.

In 2007, Nuzzi *et al.* published some model studies towards the synthesis of thymidine oligonucleotides with triazole internucleosidic linkages *via* Cu(I)-promoted ligation strategy [140]. These artificial molecular fragments are more stable than the natural products. These initial results have demonstrated a promising usefulness of such structures in search towards antisense exploitation. Also, an explorative study has been taken to prepare a trinucleotide—a triazole linked codon in a new class of oligothymidine analogues as shown by structures **9**, **10**, and **11** (Scheme 2). The authors the of work assure, that this study is a part of a longstanding research program to develop antisense nucleotides in their laboratory.

**Scheme 3:**
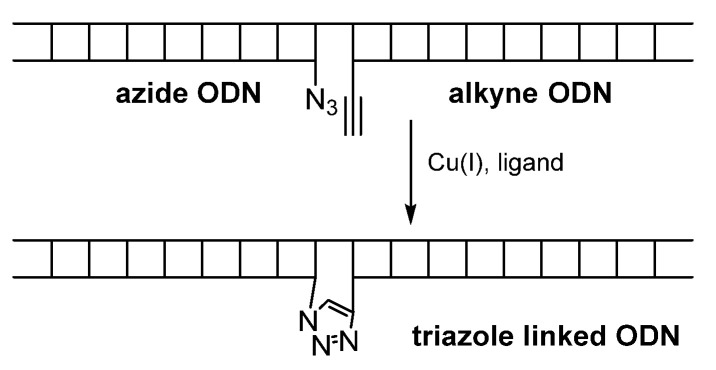
Schematic template-mediated click-ligation of two oligonucleotides.

Subsequent applications of such interesting oligomers may be found in the literature already dedicated to modified ONs. One of the first who indicated that topic, were Kumar *et al.* [141]. The click reaction is used to ligation of two single stranded oligdeoxynucleotides (ODNs), one with a 5′-alkyne and the other with a 3′-azide (Scheme 3). A covalent catenation leads to intramolecular circularization and produces a DNA strand with an unnatural extended backbone at the ligation point. The product is obtained at room temperature within 2 h.

Another exciting example of CuAAC templated nucleic acid reactions uses 3′-*O*-propargyl 5-methyldeoxycytidine at the 3′-terminus of an ODN strand, and a 5′-azide at the 5′-strand terminus [142]. As a result, the triazole linkage is similar in length to a natural phosphate-sugar linkage [143], which is tolerated by many different enzymes, such as DNA and RNA polymerases [144,145]. Thus, DNA strands with these terminal groups can be used to construct genes that are subsequently translated and transcribed in *E. coli* [116,142]. Moreover, using the CuAAC approach, epigenetic modifications can be incorporated into the gene to determine the influence of such trial [116]. This work indicates that CuAAC of 5′-azido and 3′-*O*-propargyl modifications might emerge as a non-enzymatic tool for ligation that could complement or replace ligase systems [142,146,147]. This *quick click* methodology was also used by Osman *et al.* for the same purpose—ligation of 3′-*O*-propargyl and 5′-azide DNA-modified strands through biocompatible triazole unit [148]. The CuAAC of oligonucleotides is a promising ligation system because it is as rapid but more selective than, *e.g.*, T4 DNA ligase. Triazole linkage can effectively mimic the phosphate-sugar backbone of DNA, leading to unprecedented tolerance of the ligated strands by polymerases.

**Scheme 4:**
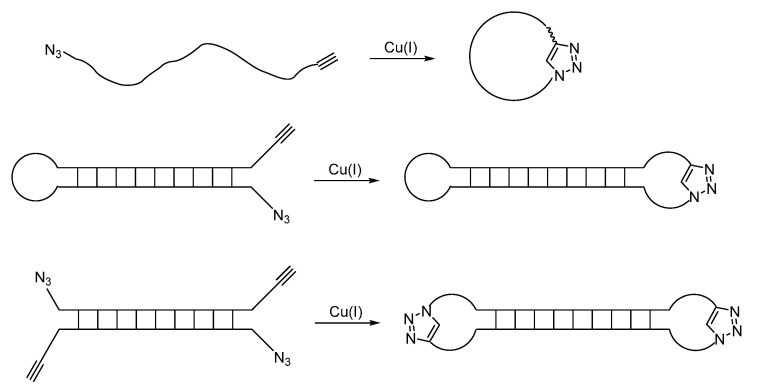
Cyclization and bicyclization of oligonucleotides.

The *click*, *bis-click*, and *stepwise click* reactions have been used for the synthesis of a covalently closed ssDNA circle and dsDNA [149,150,151] (Scheme 4). This chemistry makes it possible to receive cyclic, branched, and even bicyclic ONs [152]. Cyclization has been found to be more efficient in solution than on solid support and in both cases, it can be assisted by microwave irradiation (MW, at 60 °C for 60 min). Cyclic DNA and RNA present unusual chemical and biological properties in comparison with linear counterparts, and have been evaluated for several biological applications, including antisense, triplex, and diagnostic ones. Cyclic DNA miniduplexes are very stable, resistant to intracellular enzymatic degradation, and are more readily taken up by cells [149]. Oligonucleotide aptamers are used *in vivo* as decoys to sequester DNA-binding proteins. They must remain double stranded in the cell and be resistant to DNases. The branched cyclic ONs could be considered as mimics of a lariat structure (lariat mimics). These molecules are structurally related to natural intermediates formed during RNA splicing [153]. In turn, triazole-linked dumbbell ODNs can exist as potential decoy molecules [154]. Described triazole-cross-linked ODNs were synthesized with the use of the CuAAC and ODNs possessing *N*-3-(azidoethyl)thymidine and *N*-3-(propargyl)thymidine at the 3′- and 5′-termini. The newly synthesized dumbbell ODNs showed excellent stability against 3′-exonuclease and high thermal stability, which are necessary for decoy molecules to achieve biological responses leading to alteration of gene expression. This click methodology can be valuable in nanotechnological applications involving DNA scaffolds [155,156].

**Scheme 5:**
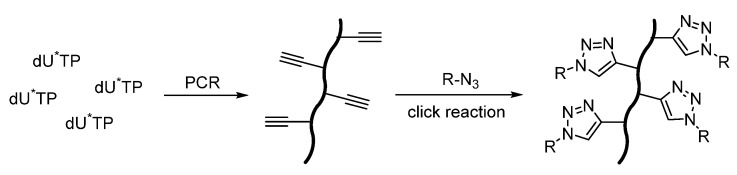
Illustrative depiction of DNA functionalization using click chemistry. R–N_3_ any azide-molecule, dU^∗^TP is a uridine derivative modified with a terminal alkyne functionality.

Another scientific idea was presented in order to investigate the efficiency of sugar labeling of DNA under various conditions [157]. For this purpose, synthetic alkyne-modified nucleoside triphosphates (dU^*^TP) were incorporated into long DNA strands by the PCR reaction, as shown in Scheme 5. Then the alkyne-modified DNA could be derivatized using the click reaction with various azide molecules, and in this case of sugars—galactose azide [158,159,160,161]. This result showed the decreased click reaction yield for DNA modified with short-chain alkyne nucleoside (ethynyluridine), rather than for DNA containing alkyne-modified uridine with an attached longer spacer. Authors also compared the click reaction efficiency of dsDNA with ssDNA 300-bases long DNA modified with 5-ethynyluridine. It turned out that both single strands, the minus- and the plus-strand, reacted in significantly higher yield compared to dsDNA. The study motivated Su *et al.* to investigate in more detail the click reaction on DNA, one of the most challenging biomolecular substrates. This work is of great practical value because labelled DNA are used in biomedical applications, for example in genetic analysis, genome screening, and high-throughput DNA sequencing. Professor Carell also included synthesis of labelled nucleotides and synthesis of probes for the sequencing of epigenetic markers [162].

**Figure 6:**
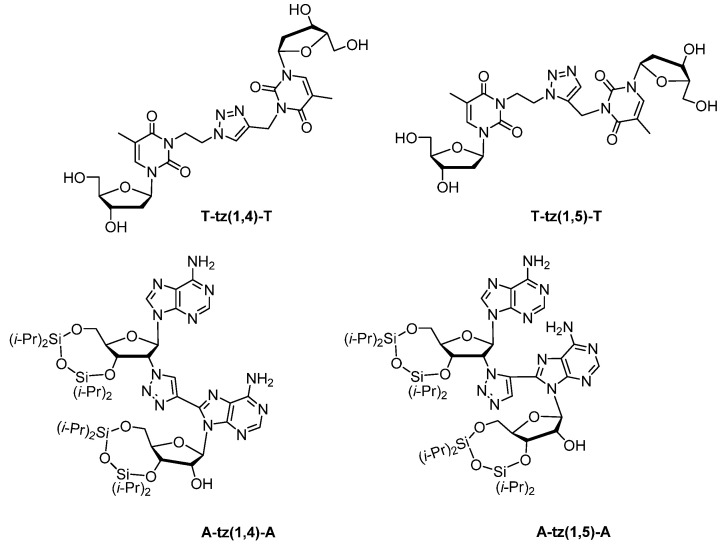
1,4- and 1,5-bisnucleosides 1,2,3-triazole derivatives of thymidine and adenosine.

The CuAAC reaction allowed for the labeling of both small synthetic oligonucleotides and large gene fragments. Interesting methods of DNA modification can be achieved in post synthetic labeling [163]. This strategy is characterized by a high degree of modularity, because changing the label does not require a novel nucleotide synthesis. The substitution of 5-position in pyrimidines or the 7-position in purines are generally well accepted by polymerases for enzymatic incorporation into PCR and primer extension products. That is why it is possible to attach alkynyl-labels, which results in DNA bearing various modifications; however, the copper ions damage DNA by breaking strands. This problem can be overcome by the use of the Cu(I)-stabilizing ligand, for example TBTA. The next limitation is that the direct incorporation of azides into synthetic DNA strands is intrinsically difficult, because the azide group reacts with the phosphorus(III) atom of the phosphoramidite group. Here, we can notice that properly prepared building blocks–nucleoside dimers may be very helpful, as new nucleic acid motifs earlier described in the literature [41,42,43,151,154,163,164,165,166,167]. Some examples of such structures are presented in Figure 6.

**Scheme 6:**
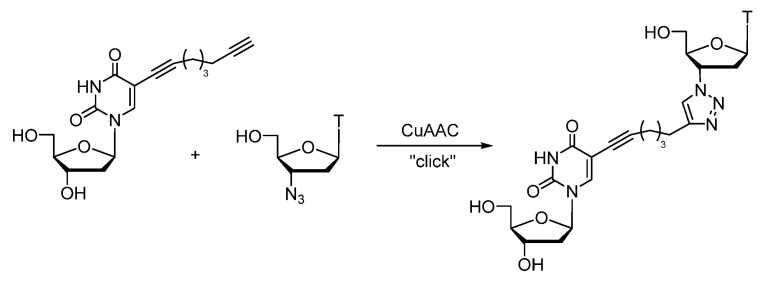
Synthesis example of a derivative containing the octadiynyl analogue of dU with AZT.

As a part of the study of modified DNA, Seela and co-workers got interested in the use of CuAAC as an efficient way to label DNA [168]. They have examined ODNs containing alkyne-labelled 7-deazapurines and pyrimidines, and later 7-alkynyl-7-deaza-2′-deoxyinosine [169]. One of such examples is presented below and applies in receiving oligonucleotides containing the octadiynyl derivative of dU with AZT *via* CuAAC reaction on solid-phase support and in solution (to link the DNA strands) [170] (Scheme 6). In their research, the fluorescence properties of oligonucleotides for the visualization of DNA was under special emphasis [171,172,173]. In particular, the generation of fluorescence by the click reaction [151,171], and studying the mechanisms of its quenching, were presented [173,174]. In turn, the introduction of a dye at the 2-position of the ribose sugar to DNA has been also done by Berndl *et al.* [175].

**Scheme 7:**
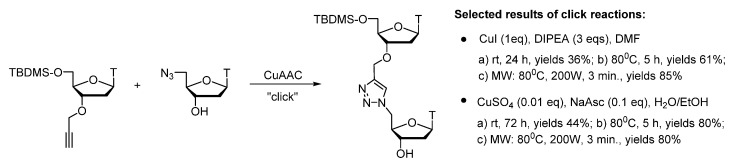
Different conditions for performing the CuAAC reaction.

The number of reports related to click chemistry and DNA is still limited, due to the required long reaction time (from hours to days), elevated temperatures (37–80 °C), and lack of any easy procedure for coupling of water-insoluble organic azides. Hence, we can notice the next step in developing click reaction (CuAAC) by using MW [176]. This highly efficient method allowed for 1,3-dipolar cycloaddition reactions to be carried out on fully deprotected ONs, affording 1,4-regioisomeric 1,2,3-triazoles in a very short time (10–15 min). The universality of the technique was also demonstrated in the synthesis of triazole-linked nucleoside dimers. First examples of such reactions have been investigated for 3′,5′-dithymidine in order to reduce the reaction time [177,178]. Zerrouki’s group reported the examination of chemical conditions for CuAAC. They tested two different salts, namely CuI and CuSO_4_, and click reactions were performed either on classical heating or microwave activation (Scheme 7). Both systems led to products, but during reaction using CuI the formation of a side product was observed at room temperature. Click reaction using CuSO_4_ gave a single product, same as in the case of classical heating (80 °C, 5 h) and MW (80 °C, 200 W, 3 min), giving identical results (80% yields). This work was continued towards tri-, tetra-, and 3′,5′-pentathymidine synthesis using this fast and efficient procedure to generate a new family of oligonucleotide analogues [178].

**Scheme 8:**
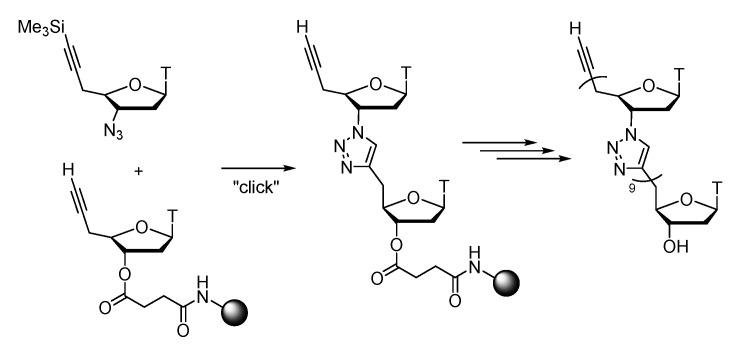
Solid-phase synthesis of 10-mer ^TL^DNA.

Further scientific reports show the research directions of triazole-linked nucleoside for longer chains design and synthesis, and even double-strand formation of such modified nucleic acids. Isobe *et al.* work on a new triazole-linked analogue of DNA using click chemistry [179]. These scientists managed to get the artificial 10-mer ^TL^DNA on a solid-phase synthesis, that after examined could form a stable double strand with the complementary strand of natural DNA (Scheme 8). The melting temperature (T_m_) of the corresponding duplex is about 40 °C higher than that of the isosequential unmodified duplex. The stability may be due to the neutral backbone that does not have any repulsive interactions with the anionic phosphate backbone of the natural target. Because similar triazole linker with a longer methylene bridge destabilizes the double strand, the six-bond periodicity of ^TL^DNA is crucial to form stable double strands. Thus, the monomer for the elongation reaction should have an acetylene group at the 5′-position and an azide group at the 3′-position. Unfortunately, this stabilization was later shown to be sequence-dependent [180]. It is worth noticing that the elongation reaction did not require an excess of the substrate, in contrast to the need in a standard phosphoramidite DNA synthesis. Additionally, microwave irradiation helps to considerably shorten the reaction time. Since the first coupling reaction proceeded smoothly and the results were encouraging, the research group improved a route for the solution-phase synthesis of 7-mer and 8-mer ^TL^DNA [180], which is aimed at preparation in a larger scale of triazole-oligomers, and even for other deoxyribonucleoside analogues bearing adenine, cytosine, and guanine nucleobases [181].

**Figure 7:**
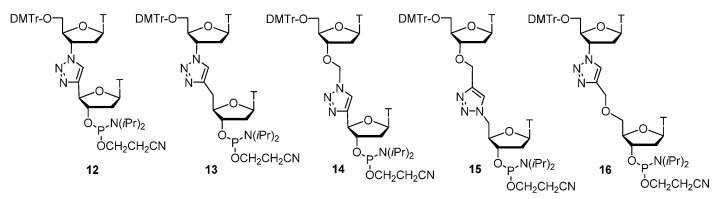
Phosphoramidite triazole-linked dimers T–T.

Chandrasekhar and Idris rightly noticed that the triazole-linkage throughout the chain is a limiting factor in the whole synthesis [182]. That is why they firstly proposed receiving a ready dimer nucleoside phosphoramidite with a triazole linkage inside (**12**) (Figure 7). Some related studies were presented by the group of Florentiev [180,183]. The group reported an improved synthesis of the dinucleoside phosporamidite block **12** and its utilization in ONs synthesis, and additionally DNA binding affinity verification. However, short linkages with four-bond triazolyl internucleotide distort the double helix, as well as the longer triazole internucleotide linkages [134,178]. It confirms that the six-bond backbone distance is crucial for the double-strand formation. The search for backbone-modified oligonucleotide analogues is still intriguing since the discovery of the CuAAC reaction. The same researchers obtained dithymidine phosphoramidite analogues with a triazole linker and an additional methylene group of type **13**, which was then incorporated in oligonucleotides using solid-phase synthesis with standard phosphoramididte protocols [180]. The hybridization ability and thermal stability of the duplexes verified destabilization effect, for which the geometry is probably responsible. Here, we can also see that the synthetic strategy based on the use of dinucleoside phosphoramidite blocks with a triazole linker is employed. The effect of single and multiple modifications on stability of mixed-base duplexes was assessed and compared with previously published data [183,184]. However, these results did not discourage the authors to continue this work, and a paper on triazole-linked oligonucleotides with high affinity to DNA complements was published a year later [185]. They obtained a new dinucleotide with a triazole internucleotide modification type **14**, that demonstrated DNA binding affinities similar to those of unmodified oligonucleotides, with simultaneous protection from nuclease hydrolysis due to the modification (Figure 7). ONs bearing these triazole fragments at the 3′-terminus, 5′-terminus or in the middle of the chain hybridized to complementary ssDNAs and did not cause any significant changes in duplex geometry. Then, the UV-melting experiments proved a slight destabilization effect of duplexes (almost 4 times lower than for other triazole modifications). Furthermore, these modified oligonucleotides were tested as PCR primers and were tolerated by polymerases. This finding shows a great potential of these new oligonucleotide analogues as the most promising triazole DNA mimics in terms of hybridization properties, compatibility with biosystems, and stability in biological liquids. The new modifications have a relatively insignificant impact on duplex geometry, therefore the authors hypothesized that it might be tolerated by ON-recognizing enzymes. It is worth noticing, that at the same time Madhuri and Kumar also worked on the same six-atom triazolide linker group of compounds **15** and **16** [186]. They synthesized two dimer blocks, then incorporated them to oligonucleotides, and UV-T_m_ with complementary DNA and RNA were presented.

**Scheme 9:**
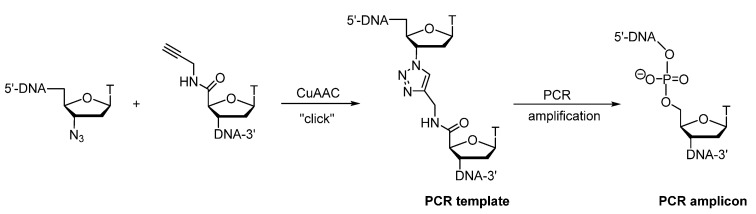
Synthesis and polymerase chain reaction amplification of DNA strands containing an unnatural triazole linkage.

At the same time, El-Sagheer and Brown worked on DNA strands containing an unnatural triazole linkage, that had been synthesized by click ligation between ONs with AZT and 4′-propargyloamido-5′-derivative of 5′-deoxymethylthymidine (Scheme 9) [187]. This is the first example of triazole-modified oligonucleotides, which serves as PCR template, which means polymerase chain reaction amplification. From the beginning it was planned, that the biological aspect of the use of these compounds is the most important here. The production of synthetic genes is prohibitive during solid-phase synthesis and it is caused by an upper limit of around 150 bases in oligonucleotides, an imperfect coupling efficiency, and chemical modifications by side-reactions during the process. So, there is a need for an inexpensive and simple method for oligonucleotide synthesis in a controlled manner and large scale. Such constructs could be used in living cells; enzyme-free synthesis of genes, which allow the incorporation of site-specific base analogues for studying DNA replication/repair; or as fluorescent dyes for in vivo visualization. Hence, the search for a polymerase-compatible triazole linkage with the initial experiments focused on non-enzymatic DNA strand ligation combined with reproducible amplification. An additional advantage of the triazole units is fact, that this chemical linkage is compatible with enzymatic processing, in particular PCR, and does not suffer from degradation due to endo- and exonucleases. It is worth noting here that DNA sequencing of the PCR amplicon and clones revealed the presence of a single thymine at the ligation site instead of the two thymine bases that were present in the original template. This is the first time that highly efficient non-enzymatic DNA strand ligation has been combined with reproducible amplification. And reverse transcription of RNA analogues containing triazole linkages will be also envisaged.

In recent years, this research group has dealt with the subject of click chemistry in the nucleic acids field in a diverse range of applications, namely: strand ligation [80,141] and cross-linking using CuAAC [188] or even receiving stable cyclic DNA miniduplexes [149]. However, the above-described experiment encouraged them to carry out further and more detailed research on the application of artificial DNA and RNA linkages in living systems [143]. So, within 10 years, they explained the correlation between structure and dynamics [189,190,191]; proved the biocompatibility of the artificial linker and the fact it could mimic the DNA phosphodiester group [144]; showed efficient RNA synthesis by a triazole-modified DNA template [145]; synthesized hairpin and hammerhead ribozymes with the triazole linkage [192]; obtained a click-linked gene that was functional in human cells [193], and thus confirmed that the importance of epigenetically modified genes and genomes increases [116]. Furthermore, they synthesized some cyclic single-stranded DNA constructs containing a single triazole, and used them as templates for RCA reaction (synthesis of the cyclic oligonucleotide) [194]. It is astonishing in the case of triazole-modified oligodeoxynucleotides, that their influence on thermodynamic destabilization of the duplexes, despite the artificial linkage (that is produced by click ligation) is read through correctly and efficiently by DNA polymerases, and is functional *in vivo*. Moreover, the resultant synthetic ribozyme with a triazole backbone mimic at the catalytic site saves its catalytic activity. This is also the first demonstration of transcription through a heavily modified DNA backbone linkage and suggestion that click-ligated DNA could be useful for the direct synthesis of biologically active RNA and proteins. Careful design has given rise to biocompatible triazole linkage that is treated as a normal one by DNA and RNA polymerases, and the gene containing triazole linkages has been shown to function in *E. coli*. This is how these scientists have pioneered a chemical method of linking DNA strands that is tolerated by living systems [144]. The artificial DNA linkage was developed using click chemistry, a highly efficient chemical reaction, to join DNA strands together without disrupting the genetic code. It means long DNA can be created quickly and efficiently by chemical methods. The genetic code could still be correctly read—this opens up all sorts of possibilities. It all means, that the fundamental genetic polymer of all living organisms on Earth, can be chemically modified to embrace novel functions that do not exist in nature.

**Scheme 10:**
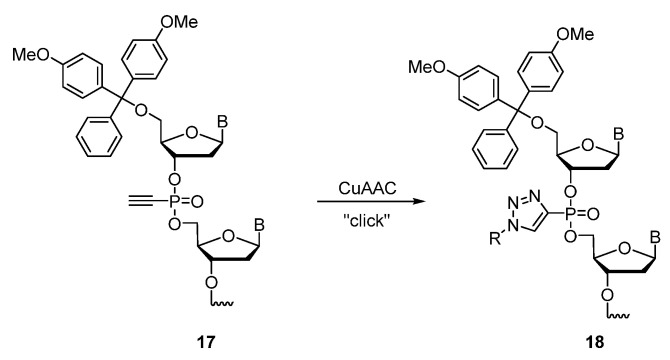
Alkynyl phosphonate DNA: a versatile clickable backbone for biological applications.

In 2012, Krishna and Caruthers had developed the 1,2,3-triazolylphosphonate internucleotide (TP) linkage, in which a 1,2,3-triazole moiety is bonded via the C4 atom to the phosphonate system [195] (Scheme 10). The resulting TP linkages 18 are formed in a two-step process. In the first step, an internucleotide linkage containing an alkyne group **17** was introduced using the classic phosphoramidite method. Such an alkynylphosphonate *clickable* backbone has versatile possibilities of biological applications. In the second step, the CuAAC reaction with selected azides was used to form a triazole system, before the synthesized oligonucleotide was detached from the solid support. In this way, it was possible to obtain oligonucleotide chimeras built from 16 to 23 mers with various modifications. Further studies allowed to state that such modified systems were resistant to 5′-and 3′-exonucleoases, and the stability of duplexes with RNA did not change significantly. The results of these experiments may encourage to continue the investigation of TP ODNs as potential antagomirs for targeting microRNA expression.

### 2.2. (TL)RNA Skeleton

The “RNA World” is various and interesting because of diversified structures and functions of ribonucleic acids [196]. In nature, RNA appeared earlier than DNA [197]. Because the DNA molecule has a more difficult accessibility in the cell, it causes many problems in gene therapy. Therefore, scientists rather use mRNA—a smaller and simpler molecule that is possible to prepare in the laboratory [198,199]. This is the medium of genetic code and acts as a pattern in protein biosynthesis. The short lifetime of mRNA, also produced for therapeutic purposes, limits its use due to enzymatic degradation in the cell. The scientists would like mRNA used in medicinal preparations to “live” longer than its natural counterpart. Of course, the biochemical and therapeutic contribution of compounds classified into the RNA family is enormous. Next to the mRNA group, a huge group of ncRNAs is present [200,201,202,203,204,205]. In particular, they are involved in drug development [206,207,208,209,210] and cancer therapy [211,212,213]. They can also act as valuable therapeutic tools in gene regulation, *e.g.*, siRNA [214], circRNA [215], miRNA [216], where triazole-modified nucleic acid elements are present in almost all described cases. In turn, the internal RNA modifications can also be achieved by the functionality transfer reaction (FTR), followed by click chemistry with a variety of clickable molecules [217]. The benefits of this method have been demonstrated by its specificity, rapidity, broad applicability, and procedure simplicity. Additionally, oligonucleotides with propargylated nucleoside as clickable moieties and a bifunctional azide have been synthesized by the CuAAC reaction [218]. This strategy has been used to obtain cross-linking in DNA–DNA and DNA–RNA duplexes for study of their stability and hybridization properties. This procedure shows how to receive 2′-*O*-propargyl-2-(amino)adenosine phosphoramidite to the formation of homodimers and heterodimers while pointing to significant stability for duplexes with terminal ligation.

Using click chemistry, small molecules are applied for creating new RNA conjugates [219,220,221], for example, alkyne-modified ATP [222] or SAM-adenosine conjugates [223]. Other, less frequent uses of triazolo-modified RNAs are applied to searching for chemical probes in nucleic acid chemistry [224,225] and in physicochemical methods like EPR spectroscopy [226] and cat-ELCCA assays [227] in biomedical sciences.

**Scheme 11:**
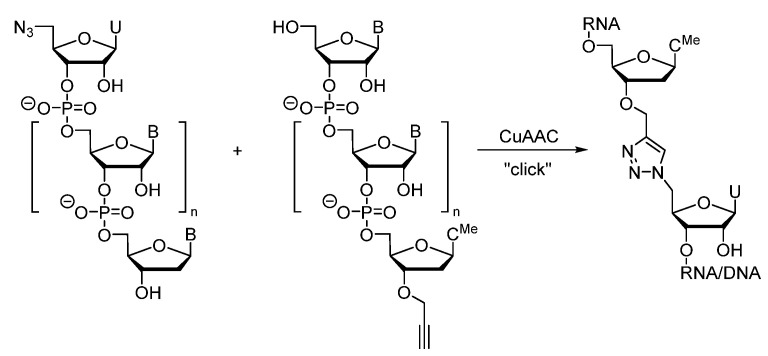
DNA–RNA hybrids obtained in CuAAC reaction.

Catalytic RNAs, named ribozymes, can catalyze cleavage or ligation of phosphodiester bonds, and many other reactions in all life forms [15]. Since nucleic acid catalysts (and aptamers) can perform additional reactions, including solutions to problems not encountered in nature, scientists have started to fathom their options. New ribozymes are being identified through a method called SELEX and can be used as therapeutics to block functions of their targets *in vivo*, or as diagnostic sensors to detect them *in vitro*. Here, the click chemistry approach comes to aid in exploring the RNA chemistry and biochemistry. An avenue to the development of new catalysts have been presented by El-Sagheer and Brown [228]. They described a strategy for the synthesis of chemically modified RNA constructs exemplified by hairpin and hammerhead ribozymes and DNA–RNA chimeras, too (Scheme 11). The CuAAC reaction was used to produce catalytically active hairpin and hammerhead ribozymes containing a novel nucleic acid backbone mimic at the catalytic site, which exhibit activity against target molecules. In the same study, the constructed DNA–RNA hybrid duplexes had the same conformation and stability as their natural counterparts. These properties gave evidence for the potential compatibility of triazole-modified oligoribonucleotides with natural nucleic acids. It proved the ability to synthesize long RNA strands by a combination of solid-phase support and click ligation, resulting in the synthesis of many biologically important RNA molecules.

A year later, the same authors presented an efficient RNA synthesis by in vitro transcription of a triazole-modified DNA template [145]. As it turned out, click chemistry has enabled the large DNA strands to produce biologically active RNA and proteins. The modified nucleic acid containing triazole linkage is correctly read-through by DNA polymerases and is functional in bacteria [144]. This work shows that T7-RNAP enzyme (T7 RNA polymerase) can transcribe through modified DNA strands to synthesize RNA, which is evidence for the surprising biocompatibility of ^TL^DNA. And this is a first example of transcription through a fully synthetic analogue of a DNA backbone. Therefore, click ligation is a strategy for overcoming the size limit of DNA and RNA obtained by solid-phase phosphoramidite chemistry (without the use of enzymes), and DNA constructs can be used directly to synthesize biologically active RNA constructs and proteins.

**Scheme 12:**
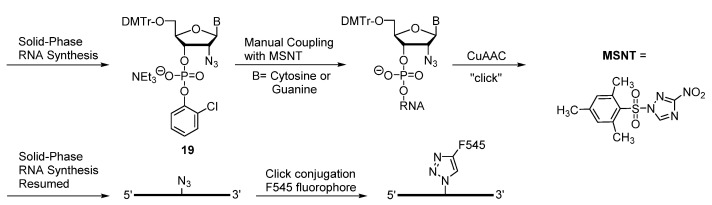
RNA synthesis using phosphoramidite chemistry in the presence of free azide moiety at C2’ position and structure of MSNT.

Chemical synthesis of RNA is less efficient than DNA owing to problems caused by the presence of the 2′-hydroxyl group of ribose, which requires selective protection during oligonucleotide assembly. This reduces the coupling efficiency of RNA phosphoramidite monomers due to steric hindrance and side-reactions that occur during the removal (or premature loss) of the 2′-protecting groups, and 3′ to 2′ phosphate migration. Additionally, the solid-phase RNA synthesis dictates the size of constructs—about 50 nucleotides in length—and longer ones remain difficult to prepare. Here, it is worth noting that most biologically important RNA molecules like ribozymes, aptamers, and riboswitches are significantly longer. Hence, a huge need for new approaches to the synthesis of RNA. The research group of Micura has reported a versatile tool for oligoribonucleotide synthesis, which consists in the introduction of clickable 2′-azido derivatives of all four canonical nucleosides as building blocks into RNA [229,230] (Scheme 12). In contrast to nucleosides modified at the 3′ and 5′ positions, compounds modified at the 2′ position can be incorporated at any position of the synthetic oligonucleotides. The only problem is the incompatibility of azides in the phosphoramidite technique (Staudinger reaction) [231]. Therefore, in most cases of oligonucleotide modification with CuAAC, nucleosides containing an alkyne substituent are introduced into the chain. However, the diester **19** can be incorporated into the formed oligonucleotide strand by the triester P(V) technique with the help of the MSNT activator [232]. The continuation of the automated synthesis did not influence the azido group and the resulting siRNA can be labeled with fluorine. As the authors claim, this work is addressed to siRNA applications and bioconjugation, where the 2′-N_3_ group is well accepted in the guide strand (antisense) and causes an increased resistance to nucleases. The rare and exceptional 2′-azido modifications are compatible with 2′-fluoro and/or 2′-*O*-methyl modifications to achieve siRNAs of rich modification patterns and tunable properties.

**Scheme 13:**
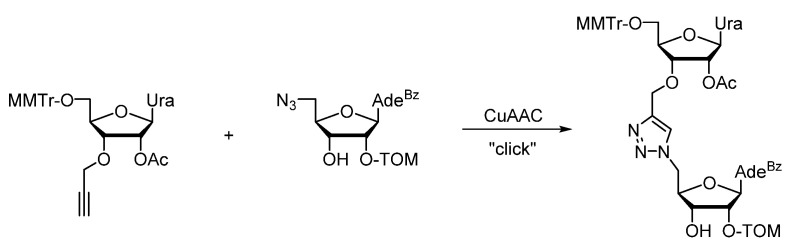
Synthesis of triazole-linked r(U_TR_A) dimer.

The synthesis and properties of triazole-linked RNA have been the subject of explorations undertaken by research groups of Rozners [233] and Isobe [234,235]. The first one made efforts towards the preparation ^TL^RNA by coupling 3′-*O*-propargyl and 5′-azidoribonucleosides followed by their study using spectroscopic techniques (Scheme 13). To evaluate the effect of triazole linkage on thermal stability of RNA, they synthesized several self-complementary sequences. The UV thermal melting analysis, CD, and NMR spectra showed that the triazole linkage strongly destabilized RNA double helix by about 7–14 °C depending on the sequence and location of the modifications. So, the triazole moiety was not a good mimic of the phosphate linkage in RNA. In contrast, Isobe showed how long triazole linkage stabilized DNA [179,184]. On the other hand, RNA tolerates better the triazole linkage than DNA, despite being two atoms longer than the phosphate [228]. The second group of scientists, after their successful work on duplex-forming ^TL^DNA, received triazole-linked trinucleotide analogues of RNA [234]. As it turned out, the synthesis of ribonucleoside analogues was more robust than in the case of deoxyribonucleoside analogues, which then underwent the elongation reaction via solid-phase synthesis using CuAAC reaction. The conditions to receive 3-mer ^TL^RNA were inappropriate for longer oligonucleotides, therefore the authors re-optimized them for 11-mer ^TL^RNA. The triazole-modified RNA was designed to explore the duplex formation, which was found to be inferior to the natural RNA oligonucleotide (in forming duplexes) [235].

^TL^DNA has been demonstrated to be a competent enzymatic substrate, but the biologically non degradable linkage of ^TL^RNA may provide an intriguing opportunity to explore the sophisticated function of RNA, for instance, RNA interference, that requires the enzymatic recognition of the oligonucleotides [234]. The design and synthesis of such constructs will certainly be investigated in the near future.

In bioconjugation chemistry the CuAAC allows for rapid labeling and ligation of RNA [236,237]. Once clickable groups are installed on RNA with free 2′-hydroxyl groups, they can be rapidly click labeled or conjugated together for biological studies. For example, triazole-linked lipid-oligonucleotide conjugates have been shown to be nontoxic, and this displays a broad applicability of click chemistry to any synthetic or natural RNA [64]. This strategy enables the capture dynamic aspects of RNA metabolism in living systems [237] and to study of the epitranscriptome marks such as new detection methods for m^6^A or mechanism-based trapping of an inosine-generating ADAR enzyme [238].

There are two major tools of biological significance in the current RNA research, namely RNA interference (RNAi) and RNA bioconjugation. RNAi is a post-transcriptional gene silencing mechanism induced by siRNA and miRNA. Here, the structural and functional repertoire of RNAs can be significantly manipulated by chemical modifications. There are high expectations for the development of siRNA and miRNA as therapeutics agents to treat diseases [229,239]. However, for applications of siRNA as therapeutic agents, chemical modification is obligatory to enhance nuclease resistance, to prevent immune activation, to decrease off-target effects, and to improve pharmacokinetic and pharmacodynamic properties. Additionally, the modifications should help these agents to penetrate cell membranes and improve siRNA delivery, and this remains one of the major challenges.

**Scheme 14:**
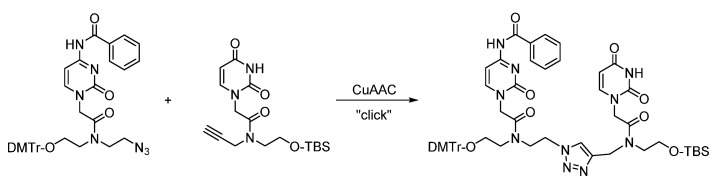
Synthesis of triazole-linked C_TR_U dimer based on a PNA-type scaffold.

Short-interfering RNAs containing various and functional spacers at the central region of the sense strand are biocompatible and suitable gene silencing substrates [240]. For example, placing cholesterol may offer a new method to be used in delivery system. The use of new functional groups and small molecules in this place allows for the creation of a new diverse class of biofunctionally active modified siRNAs, *e.g.*, cell-targeting molecules. This has enormous potential for modern medicine, and some candidate siRNAs are in clinical trials. As stated above, chemical modification of siRNAs is necessary to improve their pharmacokinetic properties. One of such methods is to generate modified siRNAs that contain triazole backbone linkages. This study is undertaken by the research group of Desaulniers, namely new siRNA design and then the evaluation of their gene-silencing potential [241,242]. Utilizing the well-known CuAAC reaction, the triazole backbone dimers (U_t_U and C_t_U) based on a PNA-like structure were synthesized and incorporated into various siRNAs targeting representative gene transcripts of an exogenous and endogenous gene (Scheme 14). The triazole-modified siRNAs were found to be capable of gene silencing of reporter genes and thus proved the compatibility as substrates within the RNAi pathway. The modifications were designed to impart potentially favorable properties to siRNAs in order to target endogenous genes, for instance the oncogene *BCL2*. This is the first example where a triazole in place of a phosphodiester backbone shows compatibility within siRNA duplexes and mimics the silencing of gene expression when positioned within the internal Watson–Crick double-stranded region of siRNAs.

Short-interfering RNAs (siRNAs) are naturally occurring biomolecules used for post-transcriptional gene regulation, so they can be promising therapeutic agents by silencing gene expression of overexpressed deleterious genes. siRNAs oligonucleotides containing commercially available 2′-modifications (2′-OMe or 2′-F) can be combined with a triazole-linked backbone modification in the siRNA design and these siRNAs are amendable within the RNAi pathway. The chemically modified siRNAs enhance gene-silencing activity and biological stability with high nuclease resistance, and keep a similar immune response as unmodified siRNAs. RNA interference might be a promising alternative to conventional chemotherapy of genetic disorders, cancer, viral infections, and other diseases for which the target mRNA can be identified. However, the short interfering RNAs (siRNAs) need to be chemically modified to be efficient therapeutic agents, in order to improve nuclease resistance, crossing of cell membranes, biodistribution, and pharmacokinetics.

**Figure 8:**
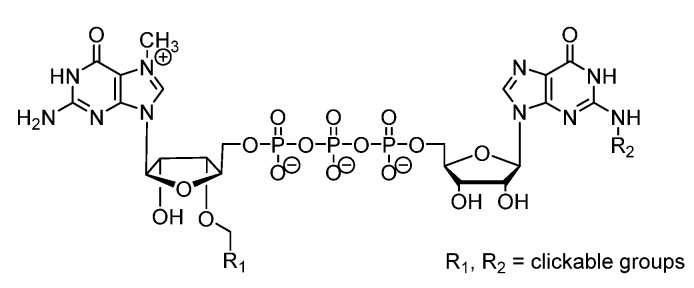
mRNA m^7^G 5′-cap clickable analogues.

Chemically modified mRNA cap structures show very promising anti-cancer properties and therefore, are being extensively studied as a target for drug discovery. A dinucleotide mRNA cap analogue obtained via click chemistry is a focus of attention for different research groups around the world. All these efforts are directed towards the improvement of intracellular delivery m^7^G caps mRNA through a cell membrane by the conjugation with cell-penetrating peptides [243], the introduction of a propargylated moiety to form a stable complex with initiation factor eIF4E [244] or for labeling strategy [245] (Figure 8). Large RNAs, such as long non-coding RNAs or mRNAs, have exciting potential as gene therapy drugs or vaccine antigens, and for such molecules post-transcriptional chemical modification will be crucial. Moreover, breakthrough technology to isolate exclusive capped mRNA opens new possibilities in the area of personalized medicine. For years, advanced research on therapeutic mRNA with a modified cap has also been conducted by Darzynkiewicz and Jemielity [246]. The modified 5′-ends of all mRNAs have been used for visualizing mRNAs in cells by fluorescent labeling, as small-molecule inhibitors of translation and/or in cancer immunotherapy and gene therapy [247,248]. The novel class of dinucleotide cap analogues containing a triazole ring within the oligophosphate chain are more durable and act with efficiency similar to their natural counterparts.

**Scheme 15:**
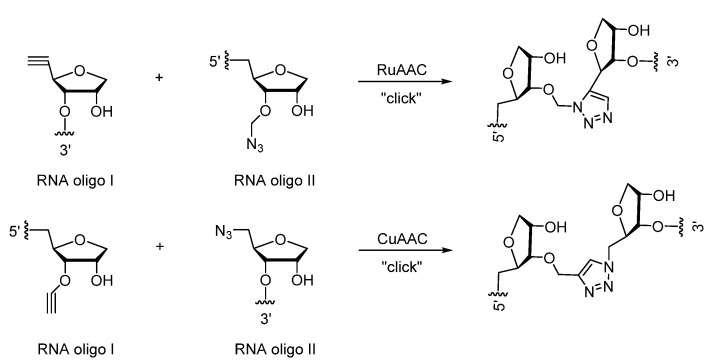
The mechanism of preparing sgRNA by click chemistry.

The Nobel Prize in Chemistry 2020 has been awarded for genetic scissors—a tool for rewriting the code of life. It is talking about the gene technology’s sharpest tools, namely the CRISPR/Cas9 genome editing method, that can change the DNA with extremely high precision [249]. This technology has revolutionized the molecular biology—it creates new possibilities for plant breeding or is contributing to new innovative therapies for cancer and inherited diseases [250]. Additionally, in this case, scientists are looking for new solutions using click chemistry approach [84] (Scheme 15). CRISPR/Cas9 system is using guide RNAs (sgRNAs) to target catalytically inactive Cas9 (dCas9) [81,83]. It gives the opportunities to study pervasive antisense transcription, namely the use of CuAAC reaction to construct DNA templates for sgRNA expression, thus generating novel sense and antisense transcripts. All this evidence confirms that stable artificial triazole DNA linker is biocompatible for PCR, read and accurately copied by DNA and RNA polymerases. Furthermore, click chemistry is an efficient method for sgRNA template construction which combined with dCas9 allows for advanced gene expression analysis. Another strategy is so-called *split-and-click* approach, where clicked sgRNAs provide easier access to individual or pools of modified sgRNAs (that are functional in cells) [82]. Click ligation of the two components generates an artificial triazole linkage that is tolerated in functionally critical regions of the sgRNA and allows for efficient DNA cleavage in vitro as well as gene-editing in cells without no unexpected off-target effects. Importantly, the site-specific incorporation of nucleotides with 2′-sugar modification or bridged nucleic acids can enhance target specificity and improve sgRNAs stability.

### 2.3. (TL)LNA and (TL)BNA Skeleton

**Figure 9:**
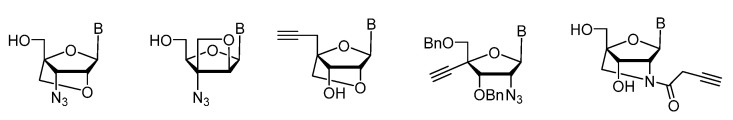
Examples of clickable monomers for LNAs and BNAs synthesis.

Locked nucleic acids (LNAs) are widely used in RNA therapeutics as LNA-modified antisense oligonucleotides [251]. From the structural point of view, LNA is an RNA derivative in which the ribose ring is constrained by a methylene linkage between the 2′-oxygen and the 4′-carbon [252,253]. This conformational restriction causes an increased binding affinity to complementary DNA and RNA sequences, but the susceptibility to RNase H should be optimized for individual applications. This provides a new chemical approach for the control of gene expression, an important consideration for antisense applications. RNase H is an enzyme that normally cleaves the RNA strand of RNA–DNA hybrids formed during lagging strand synthesis. Campbell and Wengel have found that oligonucleotides containing only LNA units form hybrids with RNA that activate RNase H to a lower degree than corresponding DNA oligonucleotides [253]. LNA units are linked by the same phosphate linkage as in DNA and RNA, allowing for synthesis of LNA oligomers using standard reagents and automated synthesizers. To be effective inside the cells or in cell extracts, antisense oligomers must be resistant to digestion by nucleases. Their therapeutic potential lies in hybridization with sequestering miRNAs—an important class of regulatory ncRNAs that can bind to partially complementary sites located in the 3′ untranslated regions (UTRs) of target mRNAs. That is why one of the most promising modifications of antisense and/or antigen oligonucleotides is the introduction of triazole or triazole-bridged nucleic acids into LNAs [254,255]. Chemical synthesis of triazole(bridged) structures also requires the intramolecular Huisgen 1,3-dipolar cycloaddition involving the ethynyl group and the azide group. It is worth emphasizing that bridged nucleic acids (BNAs) are attractive construction placed beyond LNAs and peptide nucleic acids (PNAs), with high binding affinity to ssRNA and/or dsDAN [256,257]. Novel attributes of LNAs and other classes of bridged nucleic acid analogues are extensively explored in different synthetic oligonucleotide-based therapeutics, so straightforward synthesis of their monomers could be very useful [254,255,258,259,260,261] (Figure 9).

**Scheme 16:**
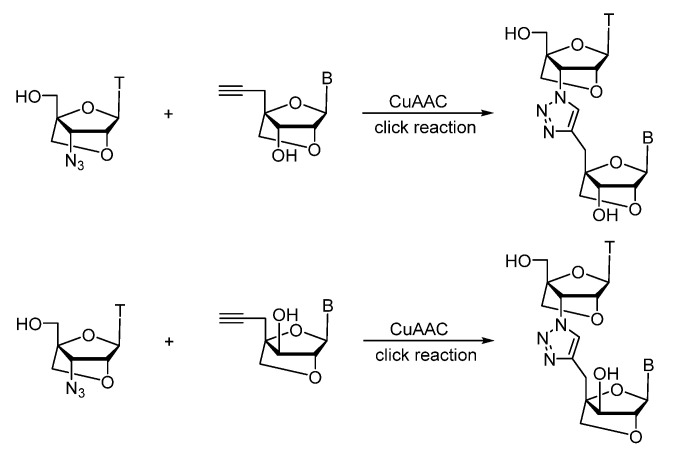
Synthesis of triazole-linked LNA-dimers using Cu(I)-catalyzed click reaction.

First attempts towards design and synthesis of LNA-based nucleoside dimers using CuAAC reaction were done in the laboratory of Prasad [262,263] (Scheme 16). In total, they received three triazole-linked LNA dimers of type T^L^–t– T^L^, T^L^–t– A^BzL^, and T^L^–t– C^BzL^. In the next step, they obtained three triazole-linked xylonucleoside dimers of type T^L^–t– T^xL^, T^L^–t– A^BzxL^, and T^L^–t– C^BzxL^. It is noteworthy to point out the effort they made in order to stich two unique nucleoside scaffolds together through a triazole ring. The optimized method can easily be adopted for automated synthesis of triazole-linked LNA-based oligonucleotides. Such structures were tested by Sharma *et al.* for their biological and biophysical properties through incorporation into gene-silencing oligonucleotides, namely antisense oligonucleotides (ASOs) and double-stranded siRNAs [260]. They presented accurate analyzes of good hybridization affinity (when incorporated at the 3′ or 5′ termini of siRNAs, but with extremely low binding affinity at internal positions), outstanding nuclease stability, and high gene silencing activity by both RNase H and Ago2 based mechanisms.

**Figure 10:**
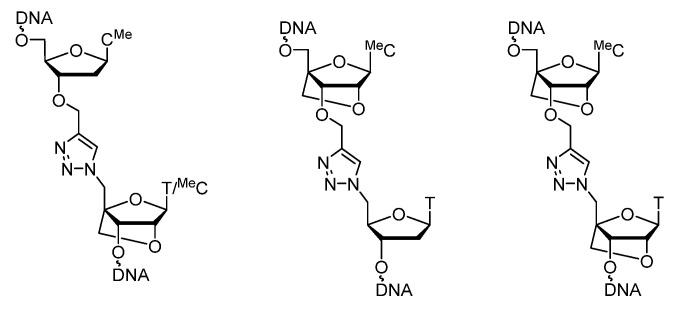
Triazole-linked DNA and LNA backbones.

Independently, Kumar *et al.* have studied the combination of triazole-linked LNAs and six-atom triazole linkage [264,265] (Figure 10). It is known that oligonucleotides containing triazole linkage form less stable duplexes with complementary RNA/DNA targets compared to unmodified DNA strands; however (TL)LNAs with internal triazole-3′-LNA linkages bind to complementary RNA with similar affinity and specificity to unmodified oligonucleotides, and the introduction of triazole moiety significantly improves the thermal stability of the modified duplex. The combined triazole-LNA linkages have demonstrated an extreme resistance to nuclease degradation (3′-exonuclease digestion). The experiment has also shown that the (TL)LNAs with LNA on either side of the triazole linkage have the highest stability against degradation by DNase, in addition to the strongest affinity for RNA targets. Thus, the combination of LNA and the triazole linkage provides a new class of potentially significant antisense oligonucleotide candidates.

### 2.4. (TL)PNA Skeleton

Peptide nucleic acids (PNAs) are oligonucleotide analogues in which the phosphodiester backbone is replaced with a polyamide structure. From a chemical point of view, PNA molecules resemble more peptides than nucleic acids. Despite the differences in relation to nucleic acids, PNA molecules with a strictly defined sequence show the ability to bind to complementary DNA or RNA molecules using Watson–Crick hydrogen bonds, blocking or reducing in this way the expression of relevant genes [266,267]. PNA is one of the most promising new molecules for recognition of nucleic acids because of their high affinity for RNA as well as to single and double-stranded DNA targets. Moreover, PNAs have received great attention due to their resistance to nuclease and protease digestion, stability in serum, and thermal stability. These compounds, due to the formation of stronger complementary bonds with DNA or RNA, were considered as excellent candidates for antisense drugs in gene therapy since their discovery [268,269]. Their disadvantage is that they are too large of molecules and it is difficult for them to penetrate inside the cell. Moreover, due to their hydrophilic nature, they are quickly excreted with urine. Therefore, prior to the introduction of such drugs, chemical modifications of PNAs or appropriate vectors should be developed to increase the bioavailability of these new antisense drugs [270,271].

**Figure 11:**
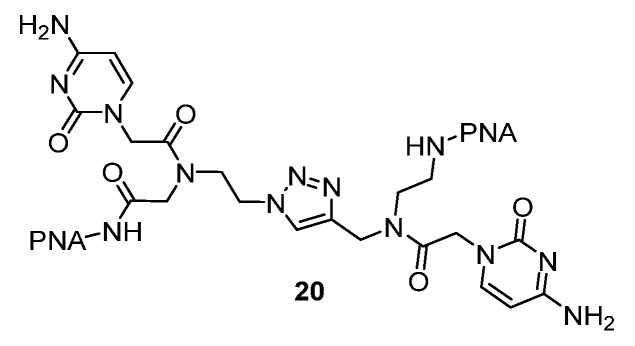
Structure of PNA linked with triazole moiety.

The research group of Wissinger has synthesized PNAs analogues bearing a triazole *in lieu* of the amide bond using a click cycloaddition [272] (Figure 11). PNA oligomers possessing an azide terminal group were obtained by standard Fmoc procedure [273], and then coupled with various alkyne monomers in the CuAAC reaction with various alkyne monomers. In the main product, dimer **20**, the modification in the form of a triazole linker shows a little effect on the hybridization ability of the modified PNA strand and the sequence accuracy of the synthesized strand. These results show that the triazole group is a good substitute for an amide linker in PNA, and such clickable peptide nucleic acids (cPNA) are an important addition in nucleic acid applications.

Among others, two research groups have published synthetic protocols for new PNA oligonucleotides that contain a triazole linkage [274,275]. The first example refers to a triazole-linked nucleoside dimer, and the second one, to a trimer. Efthymiou and Desaulniers showed a modified uracil–uracil dimer and its compatibility with hybridization to a complementary sequence when appended at the 5′ or 3′ end of DNA oligonucleotide. The second mentioned work, which has been performed in Zerrouki’s laboratory, displays the synthesis of a very interesting azido-heterotrimer, which may undergo further click reaction providing polytriazole PNA analogues. All these efforts have been made in order to synthesize chemically modified oligonucleotides with improved properties, and the results confirm that 1,4-disubstituted 1,2,3-triazole is a good mimic of a *trans*-amide bond.

### 2.5. (TL)G-Quadruplexes

G-quadruplexes (G4s) are a family of nucleic acid structures based on the formation of G-quarters of four guanines that are arranged in a plane to form several G-tetrad, stabilized by hydrogen bonds and a metal cation. G-quadruplexes represent unusual four-stranded structures found in the human DNA and RNA molecules which show unique properties including resistance to nucleases and fast folding. Interestingly, RNA and PNA also formed RNA–PNA hybrids of G-quadruplex structures [276]. Their main place of occurrence are genome regions with high transcriptional activity, the promoter regions and telomeres, so they undoubtedly take part in the regulation of gene expression.

G-quadruplexes are components of biologically important structures, they are commonly found in telomeres and oncogenes, which translates into their functions in ageing and disease development, including malignant tumors [277]. Such DNA–RNA hybrid G-quadruplex structure may also be a valuable target for anti-cancer agents directed against telomeres [278]. Telomeres are present at the ends of all eukaryotic chromosomes and play an important role in critical processes underlying genome stability, ageing, and cancer [279]. Understanding the chemistry of human telomere DNA and RNA biology will allow for the developing of chemical approaches to discover anticancer agents [280].

The related enzyme, telomerase, was first recognized as a unique and exciting anticancer target about five years after its discovery. Telomerase is activated in 80–90% of human tumors of all cancer types [281]. No other tumor-associated gene is as widely expressed as telomerase in cancers. In turn, the pioneer of DNA research on G quadruplexes, Shankar Balasubramanian, has managed to prove that formation of a G-quadruplex structure in the promoter region of the c-myc and c-kit protooncogenes inhibits their transcription and prevents the development of tumors. He has stated that in the future it will be possible to create synthetic molecules that block the proliferation of cells during the growth of cancerous tissue. So, G quadruplexes began to attract the attention of oncologists in the early 1990s, in the hope that they could help to fight cancers. The clinical experiments’ evidence for use of telomerase or telomeres as targets in cancer therapy are encouraging and are exciting prospects in future medical strategies.

**Scheme 17:**
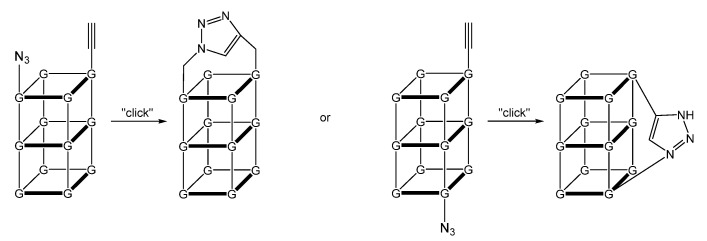
Illustrative depiction of the use of click chemistry in G-quadruplexes.

DNA–RNA hybrid G-quadruplex structure is technically difficult to study by traditional methods, such as NMR and crystallography, since various G-quadruplexes may coexist as a mixture. Click chemistry has helped overcome this difficulty. This reaction can trap a particular species or produce a snapshot of various structures that are present in a complex solution [282] (Scheme 17). The detected “azido–alkyne cycloaddition” product of the alkyne- and azido-labeled RNA and DNA coupling identifies that DNA–RNA hybrid G-quadruplex structure can be formed from human telomeric DNA and RNA sequences.

Click chemistry can be a very useful method for a search, identification, and designing therapeutics, and above all, studying the functions of these biomolecules [282,283]. DNA aptamers bearing triazole internucleotide linkages can be also found in such applications as novel thrombin-binding aptamers for anticoagulant effects [284], human telomere biology [280,285,286], and furthermore, to design and synthesize a *click-light-up* probe to investigate whether DNA-RNA G-quadruplexes exist in living cells [287]. Human telomere structures are difficult to probe in living cells because the concentration of G-quadruplexes is too low to be detected in the presence of only a few dozen chromosome ends, so employing a light-switching probe is possible to find the process of DNA–RNA G-quadruplex formation [285]. Owing to the fact that DNA aptamers are increasingly recognized as drug candidates, herein click chemistry also enables the synthesis and development of small molecules that target G-quadruplex nucleic acids to elucidate drug specificity [283].

## 3. Conclusions

In conclusion, oligonucleotides modified at the phosphodiester linkage are important class of compounds, especially in the field of antisense and antigen strategies. We are interested in the replacement of the phosphodiester linkage of the natural oligonucleotides with novel, neutral, and heterocyclic linkages based on their stability and rigidity, and therefore, report here an efficient synthesis of the 1,2,3-triazole units to prepare triazolo-modified oligonucleotides.

In this review we have demonstrated the usefulness of the CuAAC or RuAAC ligation strategy in nucleic acid chemistry. The viability of the method has been evidenced in the preparation of clickable nucleoside and nucleotide monomers, triazole-linked dimers and trimers units, and triazolo-modified oligonucleotides. The chemical modifications used in the oligonucleotide design can bypass many pharmacological shortcomings and limitations. This shows the importance of non-natural oligonucleotides acting as inhibitors of target deleterious genes responsible for various diseases. Oligonucleotides containing triazole in place of the phosphodiester linkage have been known before the area of click chemistry—the first works about the synthesis and thermal stability of triazole-linked DNA were reported by von Matt et al. But a catalyzed version of the Huisgen [3 + 2] cycloaddition, so-called click chemistry, has just enabled an intensive development of this chemistry area. The main goal of this work was to present efficient routes to design nucleic acids and their components dedicated for gene expression control. The use of gene silencing agents is a topic of intense research because these molecules can act as inhibitors of any target genes, expression of which causes diseases, among others viral infections, cancer growth, and inflammation. As naturally occurring ONs suffer from degradation due to endo- and exonucleases, modifications have become necessary to enhance the stability and improve pharmacokinetic properties from a therapeutic point of view. Modified nucleosides and nucleotides continue to attract significant focus in medical applications as building blocks for gene silencing, such as antisense, antigen, and RNA interference. The third generation of nucleic acids therapeutics—newest and most promising with enhanced binding affinity and biostability—are PNAs, LNAs, tcDNA, and CeNA.

Fundamental knowledge from many fields, including chemistry, structural biology, cell biology, and medicine, has opened up the possibility of development of novel strategies against cancer and other diseases. This review highlights the contribution of click chemistry to drug development, but also to labelling and imaging for better visualization. The click chemistry covers chemical synthesis of small molecules as well as oligonucleotides by the bio-orthogonal ligation process and their application as drugs and tools in molecular medicine. The intention of this article is to provide both inside and inspiration in the field and stimulate further research to develop new chemical approaches. Thus, click reaction is one of the most popular interdisciplinary reaction that bridges the gap between chemistry and biology.

## Data Availability

The data presented in this study are available on request from the corresponding author.

## Abbreviations

A	adenosine
Ac	acetyl protecting group
ADAR	adenosine deaminases
Ago2	argonaute RISC catalytic component 2
ASO	antisense oligonucleotide
asRNA	antisense ribonucleic acid
ATP	adenosine triphosphate
AZT	3′-azido-3′-deoxythymidine
B	heterocyclic base
BCL2	B-cell lymphoma 2 proteins
BNA	bridged nucleic acids
Bzl	benzoyl protecting group
C	cytidine
cat-ELCCA	catalytic enzyme-linked click chemistry assay
CD	circular dichroism spectroscopy
CeNA	cyclohexene nucleic acids
ceRNA	competing endogenous ribonucleic acid
circRNA	circular ribonucleic acid
cPNA	clickable peptide nucleic acids
CRISP-Cas9	clustered regularly interspaced short palindromic repeats associated protein 9
CuAAC	copper(I)-catalyzed alkyne-azide cycloaddition
CuI	copper(I) iodide
CuSO_4_	copper(II) sulfate
DIPEA	N,N-diisopropylethylamine
DMF	dimethylformamide
DMTr	4,4′ -dimethoxytrityl protecting group
DNA	2′- deoxyribonucleic acid
dsDNA	double-stranded DNA
dU	2′-deoxyuridine
dU*TP	alkyne-modified 2′-deoxyuridine triphosphate
eIF4E	eukaryotic translation initiation factor 4E
endo-siRNA	endogenous siRNA
EPR	electron paramagnetic resonance spectroscopy
eRNA	enhancer ribonucleic acid
EtOH	ethanol
FTR	functionality transfer reaction
G4	G-quadruplex
gsRNA	germline small ribonucleic acid
HCV	hepatitis C virus
HIV	human immunodeficiency virus
iPr	isopropyl protecting group
lincRNA	intervening/intergenic ribonucleic acid
LNA	locked nucleic acids
lncRNA	long noncoding ribonucleic acid
Me	methyl
min	minute(s)
miRNA	micro ribonucleic acid
mRNA	messenger ribonucleic acid
MSNT	1-(2-mesitylenesulfonyl)-3-nitro-1H-1,2,4-triazole or (2,4,6-trimethylphenyl)-(3-nitro-1,2,4-triazol-1-yl) sulfone
MW	microwave irradiation
NaAsc	sodium ascorbate
NAT	natural antisense transcript
ncRNA	non-coding ribonucleic acid
NMR	nuclear magnetic resonance spectroscopy
ODN	oligodeoxynucleotide
ON	oligonucleotide
PCR	polymerase chain reaction
piRNA	piwi-interacting ribonucleic acid
PNA	peptide nucleic acids
pRNA	promoter ribonucleic acid
rasiRNA	repeat associated siRNA
RCA	rolling circle amplification reaction
RNA	ribonucleic acid
RNAi	RNA interference
RNAi	RNA interference
RNaseP	ribonuclease P
rRNA	ribosomal ribonucleic acid
rt	room temperature
RuAAC	ruthenium(II)-catalyzed alkyne-azide cycloaddition
SAM	S-adenosyl-L-methionine
scaRNA	small Cajal body-specific ribonucleic acid
sdRNA	snoRNA-derived ribonucleic acid
SELEX	systematic evolution of ligands by exponential enrichment or in vitro selection, or in vitro evolution
sgRNA	single guide RNA
siRNA	small interfering ribonucleic acid
snoRNA	small nucleolar ribonucleic acid
snRNA	small nuclear ribonucleic acid
ssDNA	single-stranded DNA
stRNA	small temporal ribonucleic acid
T	thymidine
TBDMS	tert-butyldiphenylsilyl protecting group
TBS	tert-butyl(dimethyl)silyl protecting group
TBTA	tris(benzyltriazolylmethyl)amine
tcDNA	tricyclo-DNA
tiRNA	tRNA-derived stress-induced ribonucleic acid
(TL)BNA	triazole-modified BNA
(TL)DNA	triazole-modified DNA
(TL)LNA	triazole-modified LNA
(TL)PNA	triazole-modified PNA
(TL)quadruplexes	triazole-modified quadruplexes
(TL)RNA	triazole-modified RNA
T_m_	melting temperature
TP	1,2,3-triazolylphosphonate internucleotide linkage
tRNA	transfer ribonucleic acid
U	uridine
UTR	untranslated region of mRNA
UV	ultraviolet
